# Reduced quality of life in corneal dystrophy – a prospective case control study

**DOI:** 10.1186/s12886-025-04200-x

**Published:** 2025-06-19

**Authors:** Carolin Elhardt, Ahmad Zia Aamoon, Lennart Maximilian Hartmann, Clara Marie Sophie Grün, Armin Wolf, Christian Maximilian Wertheimer

**Affiliations:** Department of Ophthalmology, University Hospital Ulm, Prittwitzstrasse 43, Ulm, 89075 Germany

**Keywords:** Corneal dystrophy, Quality of life, Corneal densitometry, Higher order aberration, Astigmatism, Corneal pachymetry

## Abstract

**Background:**

Corneal dystrophies are a group of rare eye diseases that can cause visual impairment and affect social well-being, independence, and active participation in society. This study presents data on the impact of corneal dystrophies on quality of life compared to a healthy control group.

**Methods:**

This prospective case control study was conducted at the Department of Ophthalmology at the University Hospital Ulm in Germany. The study included 45 patients with corneal dystrophy (excluding Fuchs endothelial dystrophy) between 2021 and 2024, and a control group of 45 healthy patients. All patients completed two questionnaires on quality of life (NEI-VFQ and VF-14). The quality of life was correlated with visual acuity and Scheimpflug imaging parameters, such as higher order aberrations as well as corneal astigmatism, densitometry and pachymetry.

**Results:**

Thirteen different corneal dystrophies were included; genetic testing confirmed the dystrophies in thirteen patients (13/36 tested; 13/45 total). Mean age was 57 ± 16 years, 60% were female. Compared to the control group, patients with corneal dystrophies reported a significantly worse overall quality of life in the VF-14 (*p* < 0.001) and in all categories of the NEI-VFQ (*p* < 0.02). There was a high correlation of the quality of life in patients with corneal dystrophy with visual acuity, higher order aberrations and corneal astigmatism, and no correlation with corneal densitometry and pachymetry.

**Conclusions:**

Our data indicate that corneal dystrophies have a significant impact on an individual's quality of life in many ways. These findings highlight the importance of identifying and treating these diseases to enhance overall well-being and active participation in daily life.

## Introduction

Eye diseases can cause a wide range of visual problems that may impact an individual's ability to engage in daily activities and affect multiple aspects of their quality of life, including emotional well-being and social functioning [1]. The primary goal of treating eye diseases is to preserve or enhance visual function and quality of life [2].

During a routine examination in a clinic or practice, an estimation of the current state of ocular health can be obtained by measuring visual acuity. However, this merely provides a static measurement of the resolution of the visual processing system, with no concomitant information regarding its effect on day-to-day tasks [3]. While eye examinations and specialized tests can provide an impression of quality of vision, evaluating the patient's eye-related quality of life requires consideration of their subjective experience. This can be captured through the use of questionnaires [4]. These questionnaires measure impairment of visual function resulting from eye diseases and encompass a multitude of visual domains, including general, distance, near, peripheral, driving, and color vision. Furthermore, the assessment includes ocular pain and non-visual domains, such as general health, mental health, dependency, social functioning, and role limitations [3, 5, 6].

Corneal dystrophies are a group of rare diseases that are characterized by the presence of pathognomonic patterns of corneal deposition and morphological changes. They are typically progressive, hereditary, and non-inflammatory—although exceptions to each criterion exist [7]. They can present with a variety of signs and symptoms. As demonstrated in the literature, the effect on visual acuity [8] varies considerably ranging from excellent visual acuities in patients with significant corneal opacities to markedly reduced visual acuity [7]. However, since the effect on the impairment of life cannot be determined solely by the measured visual acuity, we assessed patients’ subjective impressions of quality of life.

To our best knowledge, there is a shortage in literature regarding patient-reported outcomes in corneal dystrophies other than Fuchs endothelial dystrophy, as most existing studies focus primarily on clinical parameters or on Fuchs endothelial dystrophy alone [9, 10]. The overall objective of this study is to examine the quality of life of patients with corneal dystrophy other than Fuchs endothelial dystrophy in order to gain insight into their needs. Moreover, the intersection between patient-reported impact of corneal dystrophies on daily life and measurable anatomical and clinical findings was investigated. This was achieved by correlating quality of life with visual acuity and higher order aberrations as well as corneal astigmatism, densitometry and pachymetry.

## Methods

### Study type

This is a prospective case control study of 45 patients with clinically diagnosed corneal dystrophy. A control group included 45 patients without any eye diseases who were matched for age and gender with the corneal dystrophy group. The data collection was performed at the Department of Ophthalmology at the University Hospital Ulm between 2021 and 2024. The study was approved by the local ethics committee of the University Ulm (approval ID: 345/20). Written informed consent from all participants was obtained prior to study participation and the procedures of the study adhered to the Declaration of Helsinki.

### Inclusion and exclusion criterion

The study included patients with a diagnosis of corneal dystrophy according to the IC3D Classification of Corneal Dystrophies, 2nd Edition [11], as determined by two corneal specialists and, in most cases, based on classic clinical appearance based on a comprehensive evaluation and diagnostic imaging. In case of disagreement, patients were not included in the study. Patients diagnosed with Fuchs'endothelial dystrophy were also excluded from the study. Forty-five healthy subjects without ocular disease, who were also examined by the cornea specialists and found to be free of ocular disease, were used as controls.

### Diagnostics and imaging

All patients underwent a comprehensive ophthalmological examination including assessment of visual acuity and slit-lamp examination following a general and ophthalmic medical history. Slit-lamp photography of the cornea was performed in all patients. OCT imaging was obtained with a spectral domain OCT (Spectralis OCT, Heidelberg Engineering, Heidelberg, Germany) in order to determine the distribution pattern of the pathological change, particularly in regard to depth within the cornea [12]. Scheimpflug imaging was conducted with the Pentacam (OCULUS Pentacam, Wetzlar, Germany). The Pentacam was utilized to obtain the following parameters: Corneal central pachymetry, corneal astigmatism, root mean squared total corneal higher order aberrations (RMS-HOA-4 mm) and corneal densitometry. For the latter, only the two inner annular zones, namely the 0 to 2 mm and the 2 to 6 mm zones, were extracted using the Cornea Densitometry Average Table of the Pentacam.

### Quality of life

The patients completed two questionnaires, the National Eye Institute Visual Function Questionnaire (NEI-VFQ) in its version with 39 questions [5] and the Visual Function Questionnaire (VF-14) [13]. The official and validated German versions of both questionnaires were used [6, 14]. The NEI-VFQ score was calculated in two distinct ways: The traditional methodology, as proposed by the NEI [15], and the Rasch [16] analysis. The traditional approach has been demonstrated to be suboptimal for the analysis of the data, but can be utilized to provide an overview of the values obtained and to facilitate comparison with other studies [15] since the use of this standard method remains quite common in literature [16]. For this purpose, the numerical values of the questionnaire were converted to a percentage scale, and the questions were grouped into 12 subscales defined by the NEI. To create a percentage value for each subscale, the responses to the questions of a subscale were averaged. A composite score was calculated by averaging all vision-targeted subscale scores, excluding the general health rating score, in order to ensure that each visual subscale was given equal weight [17]. In contrast, the Rasch analysis categorizes the questions into only two subscales, the visual function (NEI-VFQ-VF) and the socioemotional quality of life (NEI-VFQ-SE). Additionally, a score of function combining both domains is provided (NEI-VFQ-25C) [18]. This analysis has been recommended in order to reduce redundancy while maintaining the obtained information with the aim of creating simple, more valid measures and has been shown to rectify known problems with composite scores, subscale scores, missing data, and multidimensionality with the NEI-VFQ [19]. The Mann–Whitney U test was used, and results were corrected for multiple comparisons by controlling the false discovery rate using the two-stage step-up method of Benjamini, Krieger, and Yekutieli, as implemented in GraphPad Prism10.

### Genetical testing

Patients diagnosed with corneal dystrophy were offered genetic testing. Next-generation sequencing was employed to analyze the following genes, which have previously been associated with the condition: AGBL1, CHST6, COL17A1, COL8A2, CYP4V2, DCN, GRHL2, GSN, KERA, KRT1, KRT12, KRT3, LCAT, LOXHD1, MIR184, OVOL2, PIKFYVE, PRDM5, SLC4A11, STS, TACSTD2, TCF4, TGFBI, TUBA3D, UBIAD1, VSX1, ZEB1 and ZNF469.

### Statistical analysis

For data collection, Microsoft Excel 365 (Microsoft, Redmond, Washington) was used. The statistical testing was performed using SPSS (IBM SPSS Statistics, Version 28) and GraphPad Prism 10 (GraphPad Software). Statistical comparisons were performed using either a Chi square test for categorial comparison, such as biological sex distribution, or a t-test for non-normally distributed data, such as age, visual acuity, and Scheimpflug imaging parameter. For the Rasch analysis, the publicly available Excel program provided in the literature was used (version 2022–03–09) [18]. Quality of life was correlated with measurable anatomical and clinical findings and a linear relationship was tested using the coefficient of determination, denoted as *r*^2^. A *p*-value of < 0.05 was considered significant.

## Results

### Baseline characteristics and clinical presentation

The following 13 different corneal dystrophies in 45 patients were included: 12 × epithelial basement membrane corneal dystrophy, 1 × epithelial recurrent erosion dystrophy, 1 × Lisch corneal dystrophy, 1 × gelatinous drop-like corneal dystrophy, 5 × Reis-Bucklers corneal dystrophy, 5 × lattice corneal dystrophy, 9 × granular corneal dystrophy (2 × type 1, 7 × type 2), 4 × Schnyder corneal dystrophy, 2 × macular corneal dystrophy, 1 × Fleck corneal dystrophy, 1 × posterior amorphous corneal dystrophy, 3 × posterior polymorphous corneal dystrophy. An equal number of patients were included in both the corneal dystrophy and the control group, with no statistically significant differences in age (*p* = 0.052) and biological sex distribution (*p* = 0.069). Patients with corneal dystrophy had significantly worse visual acuity, higher pachymetry, higher astigmatism, higher total corneal higher order aberrations and higher corneal densitometry of the full thickness cornea in both the 0–2 mm and the 2–6 mm zones (Table 1). Of the 45 patients clinically diagnosed corneal dystrophy, 36 (80%) consented to genetic testing for corneal dystrophy. In 13 patients only, the corneal dystrophy was genetically confirmed and thereby supporting the clinical diagnosis.

**Table 1:** Differences in corneal dystrophy compared to control. Chi-square test used for biological sex distribution and independent t-test for all other comparisons. (SD = Standard deviation)

	**Corneal dystrophy (Mean** ± **SD)**	**Control (Mean** ± **SD)**	***p*-value**
Patients included: *n* = 45 per group			
Age (years)	57 ± 16	52 ± 11	0.052
Biological sex (female)	27 (60%)	35 (78%)	0.069
All (better and worse) eyes included: *n* = 90 per group			
Visual acuity (logMAR)	0.43 ± 0.43	0.0 ± 0.04	< 0.001
Pachymetry (µm)	580 ± 80	551 ± 32	0.001
Corneal astigmatism (dpt)	1.65 ± 1.77	0.82 ± 0.60	< 0.001
Total corneal higher order aberration (4 mm) (µm)	0.6 ± 0.7	0.1 ± 0.04	< 0.001
Densitometry 0–2 mm zone	28 ± 12	18 ± 1	< 0.001
Densitometry 2–6 mm zone	25 ± 10	18 ± 2	< 0.001

### Quality of life

**Fig. 1:**
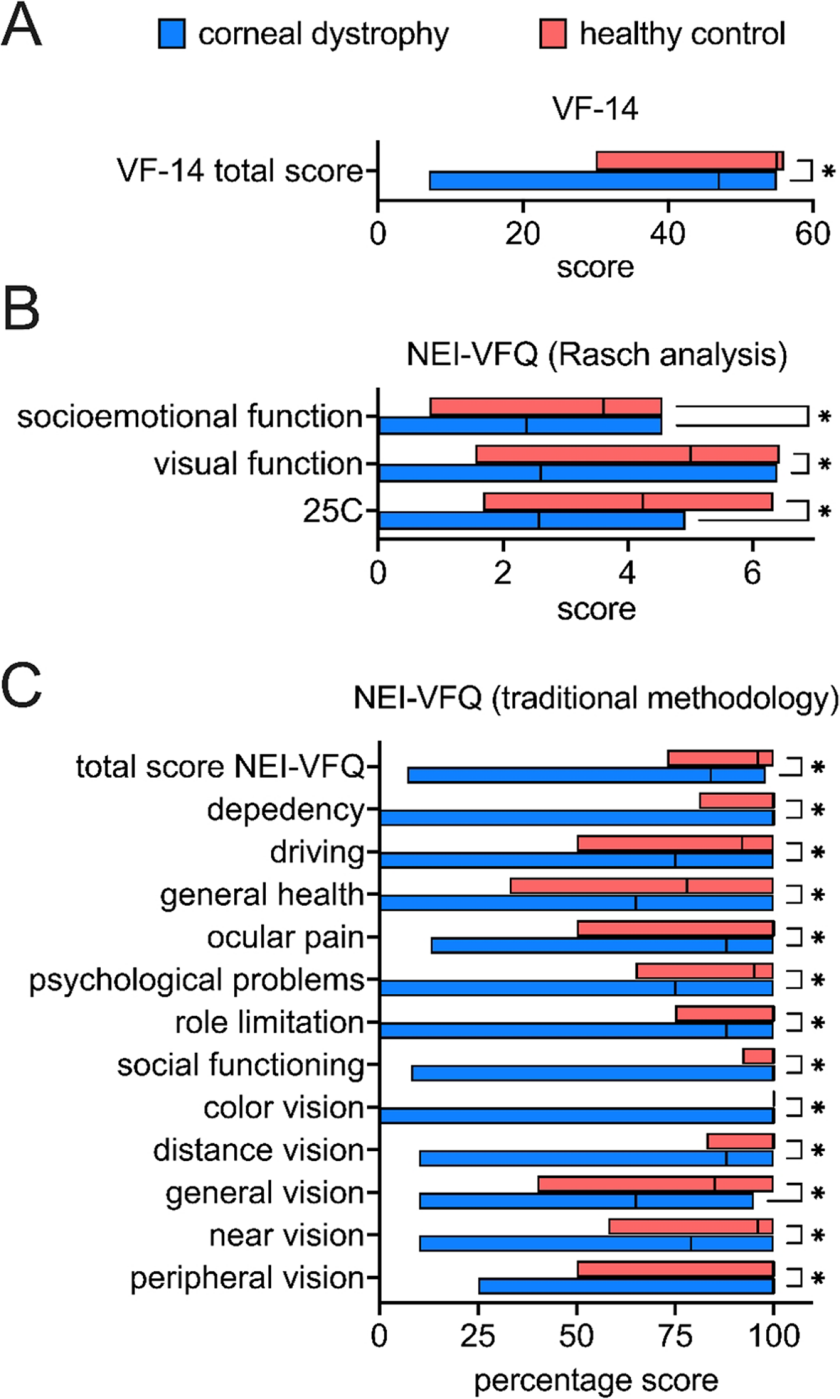
Quality of life in corneal dystrophy. The quality of life of the patients in this study was measured with the above-mentioned questionnaires VF-14 and NEI-VFQ and was significantly reduced in all comparisons. An asterisk (*) in the box indicates a significant discovery after correcting for false discovery rate in multiple testing

Quality of life of the patients in this study was assessed using the above-mentioned questionnaires VF-14 and NEI-VFQ and was significantly reduced in all comparisons. Specifically, patients with corneal dystrophy reported worse quality of life than the control group in both questionnaires (VF-14: *p* < 0.001, NEI-VFQ: *p* < 0.001). In the traditional subgroup analysis, the patients with corneal dystrophy showed lower scores in all subcategories of the NEI-VFQ: general health (*p* = 0.02), general vision (*p* < 0.001), ocular pain (*p* = 0.002), near vision (*p* < 0.001), distance vision (*p* < 0.001), social functioning (*p* < 0.001), psychological problems (*p* < 0.001), role limitation (*p* < 0.001), dependency (*p* < 0.001), driving (*p* < 0.001), color vision (*p* = 0.01) and peripheral vision (*p* < 0.001). Similarly, Rasch analysis demonstrated significantly lower scores in patients with corneal dystrophy compared to the control group in both subcategories, NEI-VFQ-VF (*p* < 0.001) and NEI-VFQ-SE (*p* < 0.001), as well as in the score of function NEI-VFQ-25C (*p* < 0.001) which combines visual function and socioemotional domains (Fig. 1). All comparisons reached statistical significance and reported as discovery after correction for false discovery rate in multiple testing.

### Correlation of quality of life to visual acuity and imaging

**Fig. 2:**
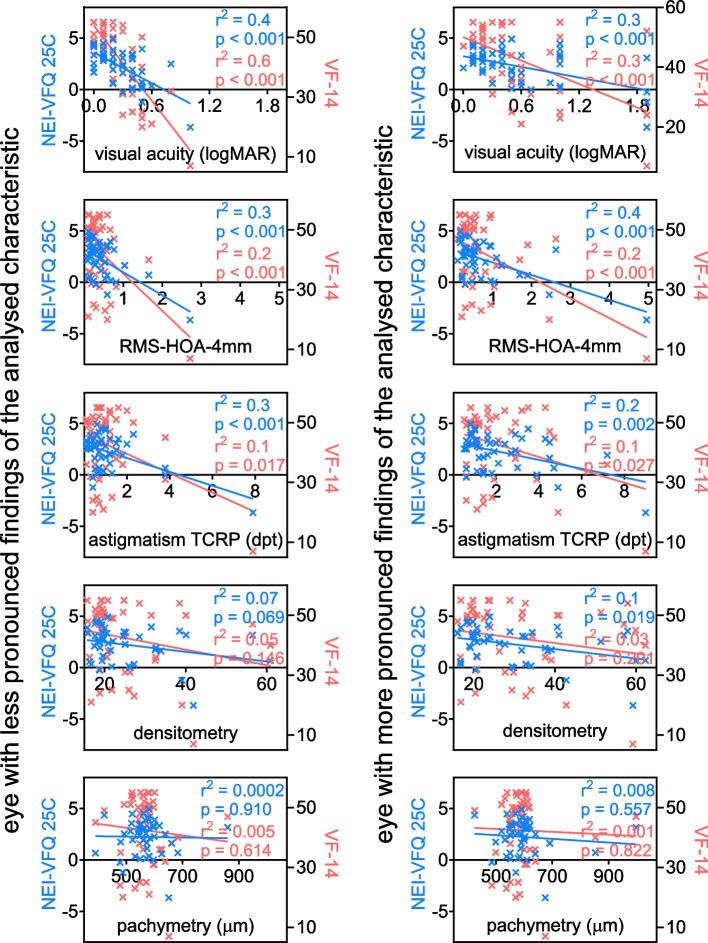
Correlation between quality of life and clinical parameter. A linear regression analysis was conducted to examine the relationship between the compound scores of the questionnaires VF-14 and NEI-VFQ-25C and visual acuity, as well as Scheimpflug imaging parameters such as astigmatism, higher order aberrations, corneal densitometry, and corneal pachymetry. The analysis revealed an inverse correlation between quality of life and visual acuity, higher order aberrations, and astigmatism, with a statistical significance that the slope is non-zero and a good coefficient of determination. (RMS-HOA = higher order aberration; VF-14 = Visual Function Questionnaire 14; NEI-VFQ-25C = National Eye Institute Visual Function Questionnaire)

The quality of life in corneal dystrophy patients was correlated with visual acuity and Scheimpflug imaging parameters such as higher order aberrations, corneal astigmatism, corneal densitometry, and corneal pachymetry. This method was applied to the eye exhibiting the less pronounced findings of the analyzed characteristic, and to the contralateral eye with the more pronounced characteristic. An inverse correlation of quality of life with a slope statistically significantly different from zero and a good coefficient of determination was found for visual acuity, higher order aberrations and astigmatism for both the better and worse eye and for both of the questionnaires used (Fig. 2). The slope of the linear regression between the quality of life results and both densitometry and pachymetry in both eyes was not statistically significantly different from zero and the correlation coefficient was low.

## Discussion

Vision-related quality of life is a significant factor in the evaluation of disease burden and the efficacy of ophthalmological interventions [20]. Our study examined the quality of life in patients with corneal dystrophy. We found that several types of the disease had a substantial impact on an individual's quality of life in various aspects of daily activities. The reduction in quality of life was found to be inversely correlated with logMAR visual acuity or higher order aberration.

Visual acuity is a straightforward method for quantifying visual performance. However, patients are primarily concerned with the vision-related quality of life they experience as a result of their functional visual performance [19]. It is not solely central vision that determines one’s ability to manage daily activities; overall visual function also plays an important role. This has been clearly demonstrated, for instance, in glaucoma patients, who may retain intact central vision yet still report an impaired quality of life [21]. Nevertheless, in our study group, even the central visual acuity exhibited strong correlations with nearly all aspects of quality of life. However, other clinical findings such as higher order aberrations and corneal astigmatism also correlated with the quality of life. It is well documented that higher order aberrations can affect visual function and consequently, they warrant attention, especially when assessing the impact of corneal changes on visual performance [22].

A review of the relevant literature revealed a notable lack of studies that directly examined the quality of life in corneal dystrophy. Existing research, as far as could be ascertained, focuses, for example, on improvements in visual acuity and other objective parameters, such as corneal irregularities and the degree of opacities in the cornea following surgical procedures [9, 23]. Objective improvements are sometimes used to infer an enhancement in quality of life; however, patient’s quality of life is not directly assessed [24]. One exception is Fuchs endothelial dystrophy, for which a decreased quality of life was demonstrated, which improved after keratoplasty as measured by the NEI-VFQ [10, 25]. Furthermore, other studies have reported a reduction in the quality of life for example in families with corneal lattice dystrophy [26]. Our data align with these results; however we deliberately excluded Fuchs dystrophy from our analysis, as its impact on quality of life has already been well documented in the literature [7].

According to the Lancet Global Health Commission, eye health has a profound influence on quality of life, including general health, well-being and social integration [27]. This was reflected in our study of patients with corneal dystrophies: Patients reported not only vision-related difficulties, but also an impact on non-visual aspects of their lives. The disease affected both physical health and mental health, dependency on others, social functioning and role limitations. Therefore, our study showed that corneal dystrophies could reduce quality of life in several ways – both visual and non-visual. Given the well-established impact of visual impairment on the health care system, it is important to also consider the economic implications [27]. Furthermore, for patients whose vision-related quality of life is markedly reduced and affects their independence, access to supportive services—such as low vision rehabilitation, psychological counseling, or social support—should be actively promoted as an integral component of comprehensive patient care.

In a population-based study both eyes were labeled as better and worse eye according to their logMAR visual acuity. It was shown that the degree of visual impairment in the worse eye had a greater impact on quality of life than in the better eye [28]. Similar findings were reported in glaucoma, where the worse eye—in regard to the visual field—was more strongly associated with a reduction in everyday visually-guided actions [29]. The results of our study could not confirm these findings. However, we were able to show that abnormal refractive changes of the corneal surface and overall visual acuity were more determining for quality of life than structural findings like opacification in terms of light backscatter in Scheimpflug densitometry and corneal pachymetry. This might have clinical relevance for the treatment of certain corneal dystrophies with the excimer laser, where complete removal of opacities is frequently not possible, but the flattening of the surface is of benefit to the patient.

The corneal dystrophies included in this study present a spectrum of disease and corneal opacity, manifesting in various forms. It has been described that these changes affect the light propagation into the eyes, potentially affecting visual acuity and quality [30]. Furthermore, it is known that corneal lesions can lead to decreased sensitivity to contrast and to increased sensitivity to glare [31]. Corneal changes such as corneal infiltrates [32] or corneal deposits [33] can result in increased light backscattering. A study on granular corneal dystrophy reported higher order aberrations that were associated with the disease [34]. Higher order aberrations are well documented to negatively impact visual performance and contrast sensitivity [22]. Therefore, they deserve particular attention, particularly when assessing the impact of corneal changes on visual performance.

In the majority of cases, a specific corneal dystrophy can be diagnosed based on clinical examination alone [7]. However, it is important to consider the possibility of genetic testing, particularly to confirm the diagnosis or differentiate between similar types of dystrophies. In our study, 13 of the patients were found to have a genetically detectable corneal dystrophy. Additionally, the referral of a patient with Schnyder corneal dystrophy from our eye clinic to the department of human genetics led to the examination of family members resulting in the identification of the genetic mechanisms responsible for the condition in several relatives. This highlights the added value of genetic analysis for the diagnosis of corneal dystrophies.

It is important to acknowledge that quality of life assessments are inherently subjective, and the generalizability of these results to all patients is limited. The perceived impact of disease severity may vary depending on individual factors such as their occupation, hobbies, and age of onset. The objective of our study was to derive broader conclusions by including a wide age range and a spectrum of different corneal dystrophies. A limitation of this study lies in the relatively small number of patients within each individual dystrophy group, which reflects the rarity of these conditions. The heterogeneity of the included corneal dystrophy subtypes, combined with the limited sample sizes per group, precluded meaningful subgroup analyses. As the primary aim was to assess the overall impact of corneal dystrophies on vision-related quality of life, the study was not powered to detect differences between specific dystrophy forms. Therefore, no subgroup analyses were performed, and no conclusions can be drawn for individual dystrophies based on the present data. Further studies with larger cohorts are needed to enable robust subtype-specific investigations. Another limitation is the limited number of genetically confirmed cases potentially resulting from a limited genetic test confined to certain genes and the not well-defined genetic inheritance in certain corneal dystrophies. All included cases were diagnosed based on comprehensive clinical evaluation and multimodal imaging by experienced corneal specialists, which represents the current diagnostic standard in clinical practice. Where available, genetic testing served as a complementary tool to support the accurate classification of certain cases into the appropriate dystrophy subtype.

In conclusion, our data indicates that corneal dystrophies can profoundly affect patients' daily lives in a variety of ways. Understanding the impact of a disease on patients' quality of life is essential for making appropriate clinical decisions and improving patient well-being. Clinicians should be encouraged to not only assess clinical parameters but also actively address reduced quality of life in their therapeutic approach, including patient counseling and management planning. The patient's treatment regime should be discussed with the patient in question, taking into account their expectations, current level of suffering, perceived quality of life, and expected improvements in vision and overall well-being.

## Data Availability

The data that support the findings of this study are not openly available due to reasons of sensitivity and are available from the corresponding author upon reasonable request.

## Abbreviations

NEI-VFQ: National Eye Institute Visual Function Questionnaire
NEI-VFQ-SE: Socioemotional quality of life score of the National Eye Institute Visual Function Questionnaire
NEI-VFQ-VF: Visual function score of the National Eye Institute Visual Function Questionnaire
NEI-VFQ-25C: Score of function of the National Eye Institute Visual Function Questionnaire
OCT: Optical coherence tomography
VF-14: Visual Function Questionnaire
